# Supplementary material for: Yiai Fuzheng decoction inhibits triple-negative breast cancer by remodeling the immune microenvironment
Source: Front Immunol. 2025 Sep 30;16:1615631. doi: 10.3389/fimmu.2025.1615631 (PMC12518410; doi:10.3389/fimmu.2025.1615631)
Supplement: Supplementary file 2 [file DataSheet1.zip › Supplementary File 2/Treatment/D_vs_H/07_Pathway_Analysis/Pathway_Analysis/Sig_Pathways_ID/Glutathione metabolism.pdf]

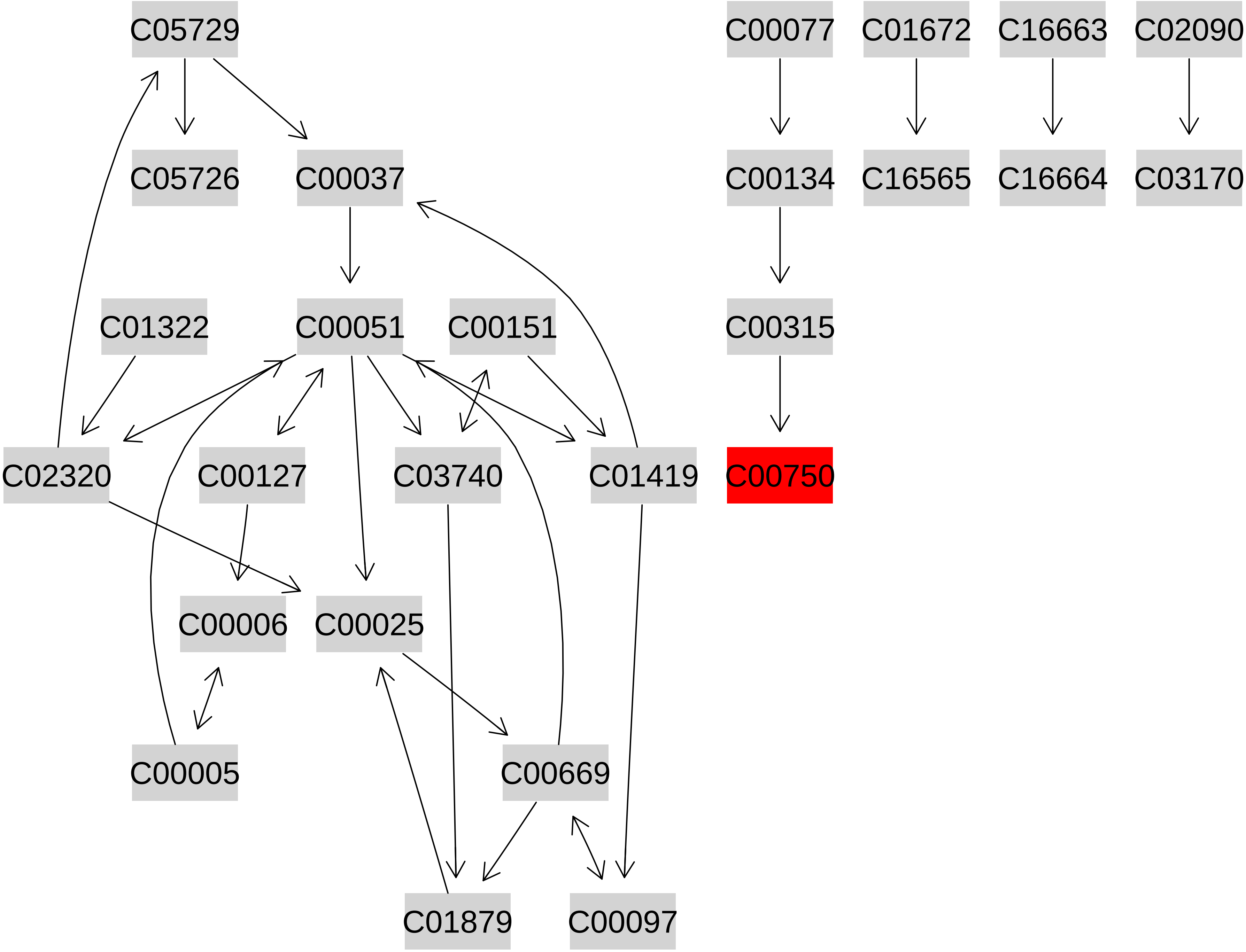

C05729
C00077
C01672
C16663
C02090
C05726
C00037
C00134
C16565
C16664
C03170
C01322
C00051
C00151
C00315
C02320
C00127
C03740
C01419
C00750
C00006
C00025
C00005
C00669
C01879
C00097
